# Kinetochore-catalyzed MCC formation: A structural perspective

**DOI:** 10.1002/iub.2697

**Published:** 2022-12-14

**Authors:** Elyse S. Fischer

**Affiliations:** MRC Laboratory of Molecular Biology, Cambridge Biomedical Campus, Cambridge, UK

**Keywords:** Bub1, Cdc20, Mad1, mitotic checkpoint complex, Mps1, spindle assembly checkpoint

## Abstract

The spindle assembly checkpoint (SAC) is a cellular surveillance mechanism that functions to ensure accurate chromosome segregation during mitosis. Macromolecular complexes known as kinetochores, act as the interface of sister chromatid attachment to spindle microtubules. In response to unattached kinetochores, the SAC activates its effector, the mitotic checkpoint complex (MCC), which delays mitotic exit until all sister chromatid pairs have achieved successful attachment to the bipolar mitotic spindle. Formation of the MCC (composed of Mad2, BubR1, Bub3 and Cdc20) is regulated by an Mps1 kinase-dependent phosphorylation signaling cascade which assembles and repositions components of the MCC onto a catalytic scaffold. This scaffold functions to catalyze the conversion of the HORMA-domain protein Mad2 from an “inactive” open-state (O-Mad2) into an “active” closed-Mad2 (C-Mad2), and simultaneous Cdc20 binding. Here, our current understanding of the molecular mechanisms underlying the kinetic barrier to C-Mad2:Cdc20 formation will be reviewed. Recent progress in elucidating the precise molecular choreography orchestrated by the catalytic scaffold to rapidly assemble the MCC will be examined, and unresolved questions will be highlighted. Ultimately, understanding how the SAC rapidly activates the checkpoint not only provides insights into how cells maintain genomic integrity during mitosis, but also provides a paradigm for how cells can utilize molecular switches, including other HORMA domain-containing proteins, to make rapid changes to a cell's physiological state.

## Abbreviations

APC/C: anaphase-promoting complex/cyclosome
C-Mad2: closed-state Mad2
CTD: C-terminal domain
FRET: fluorescence resonance energytransfer
KEN: Lys-Glu-Asp
MCC: mitotic checkpoint complex
MIM: Mad2-interacting motif
NTD: N-terminal domain
O-Mad2: open-state Mad2
PDB: protein data bank
RLK: Arg-Leu-Lys
SAC: spindle assembly checkpoint
TPR: tetratricopeptide repeat

## 1 Introduction

**Figure 1:**
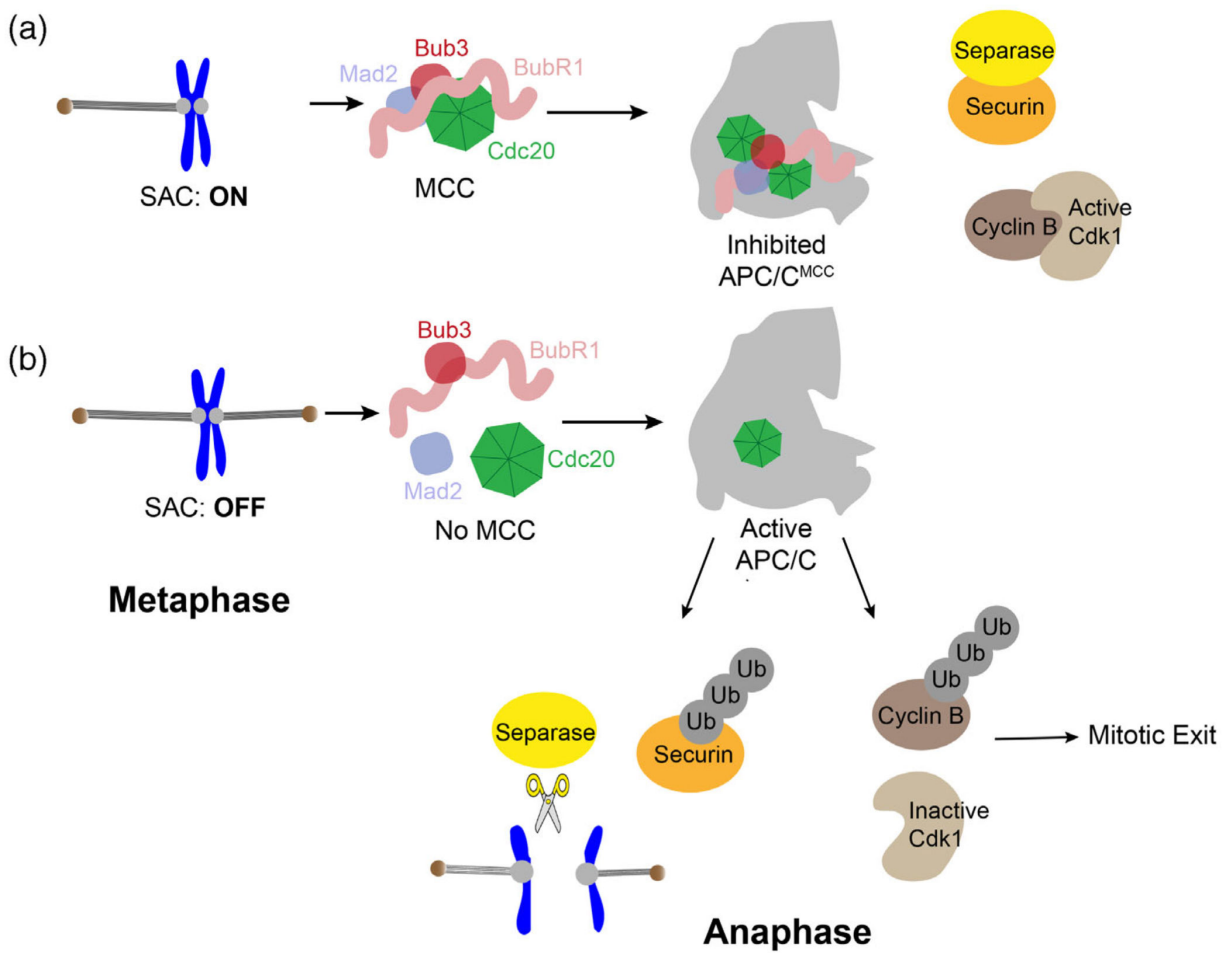
Overview of the Spindle Assembly Checkpoint (SAC). (a) During metaphase, at unattached kinetochores, the SAC activates its diffusible inhibitor, the mitotic checkpoint complex (MCC), which binds and inhibits the anaphase-promoting complex/cyclosome (APC/C) to prevent premature securin and cycle B degradation. (b) Upon biorientation of kinetochore-microtubule attachments, SAC signaling is turned off, the MCC is disassembled, and the APC/C is free to ubiquitinate securin and cyclin B to trigger anaphase onset and subsequent mitotic exit.

The accurate segregation of sister chromatids to daughter cells during the metaphase-to-anaphase transition of mitosis is critical to maintaining the faithful inheritance of genetic material and genomic stability.^1,2^ Segregation errors result in genomic abnormalities, including a loss or gain of a chromosome, known as aneuploidy, which ultimately leads to cell death and/or disease.^1–3^ Chromosome segregation is safeguarded by the spindle assembly checkpoint (SAC).^4–9^ This process is mediated by large proteinaceous assemblies known as kinetochores which are assembled onto the core of centromeric chromatin and act as the interface for microtubule attachment to sister chromatids.^3,8,10,11^ Kinetochores are also the signaling hub required to create communication between the state of microtubule attachment and SAC activity.^12^ Unattached kinetochores trigger activation of the SAC, which creates a “wait for anaphase signal,” halting anaphase onset until all kinetochore-microtubule attachments are made (Figure 1a).^13,14^

The SAC delays anaphase onset by generating a diffusible inhibitor known as the mitotic checkpoint complex (MCC), comprised of two subcomplexes, BubR1: Bub3 and Cdc20:Mad2 (Figure 1a).^13–15^ The BubR1:Bub3 complex remains stable throughout mitosis.^16–19^ In contrast, Cdc20:Mad2 association involves a kinetically disfavored major structural rearrangement of Mad2 from an “inactive” open-state (O-Mad2) into an ‘active’ closedstate (C-Mad2), catalyzed by unattached kinetochores.^20–23^ Once formed, the MCC binds and inhibits an E3 ubiquitin ligase, the anaphase-promoting complex/cyclosome (APC/C), whose ubiquitination activity is required to target cyclin B and securin for degradation (Figure 1a).^24–31^ Once all sister chromatid pairs have achieved successful attachment to the bipolar mitotic spindle, the SAC must be silenced to relieve APC/C inhibition and allow timely mitotic exit (Figure 1b).^4,5,32,33^ This includes kinetochore-based SAC-silencing mechanisms such as the phosphatase activity of protein phosphatase 1 (PP1) and protein phosphatase 2 B56 (PP2-B56), dynein-mediated stripping of SAC components, as well as MCC-specific silencing mechanisms including TRIP13-mediated disassembly of the MCC, and MCC ubiquitination by the APC/C.^4,5,32,34–43^

The structure and function of the MCC and how it binds and inhibits the APC/C are well established.^24–28,44^ More elusive has been uncovering how the SAC overcomes the kinetic barrier to Cdc20:C-Mad2 association to rapidly produce the MCC. Recently, several new studies, building on decades of experimental work, have unveiled a compelling model for how this is achieved.^5,23,45–49^ Unattached kinetochores activate an Mps1-dependent phosphorylation signaling cascade which sequentially recruits checkpoint proteins to the outer kinetochore to assemble a catalytic scaffold for MCC formation.^5,45,47^ The scaffold functions to not only locally concentrate but also promote the accessibility and spatial repositioning of Cdc20 and Mad2 to catalyze their interaction.^5,46,48,49^ This review will first overview the structure and function of the MCC (Section 2) and the molecular mechanisms underlying the kinetic barrier to its formation (Section 3). After which, how Mps1 orchestrates the assembly of the catalytic scaffold will be outlined (Section 4). Our current understanding of the structure of the catalytic scaffold (Section 5) and how it acts to catalyze the Cdc20:C-Mad2 association (Section 6) will then be presented and discussed.

## 2 Mcc

### 2.1 Architecture of the MCC

**Figure 2:**
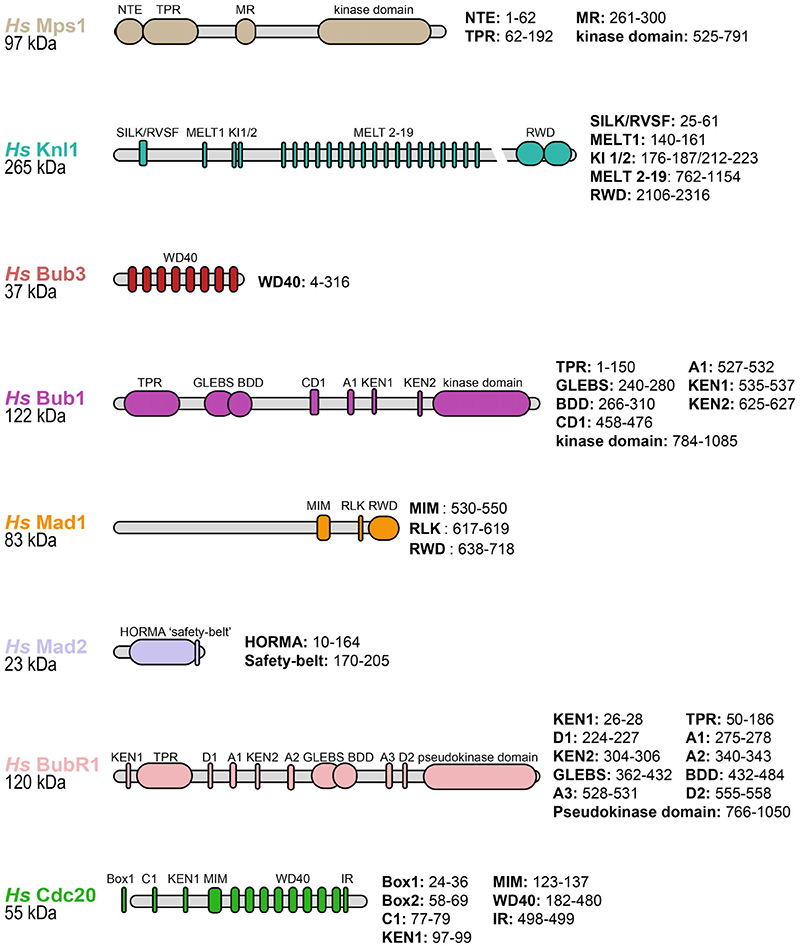
A schematic diagram of mitotic checkpoint complex (MCC) subunits and key proteins required for MCC assembly onto the outer kinetochore. Functional domains are outlined in colored ovals and their residues numbers are outlined to the right of each schematic. NTE: N-terminal extension. MR: Middle region. TPR: Tetratricopeptide repeat. SILK/RVSF: Ser-Ile-Leu-Lys/Arg-Val-Ser-Phe. MELT: Met-Glu-Leu-Thr. KI: Lys-Ile. RWD: RING, WD40, DEAD. WD40: Trp-Asp 40. A: ABBA (named ABBA as the motif was originally identified in A-type cyclins, Bub1, BubR1 and Acm1, though it has also been referred to as the Phe motif). GLEBS: Gle2-binding-sequence. BDD: Bub dimerization domain. KEN: Lys-Glu-Asn-box. CD1: Conserved domain 1. MIM: Mad2-interacting motif. RLK: Arg-Leu-Lys. HORMA: Hop1p, Rev7p, Mad2 proteins. C: C-box. D: Destruction box. IR: IR-tail.

**Figure 3:**
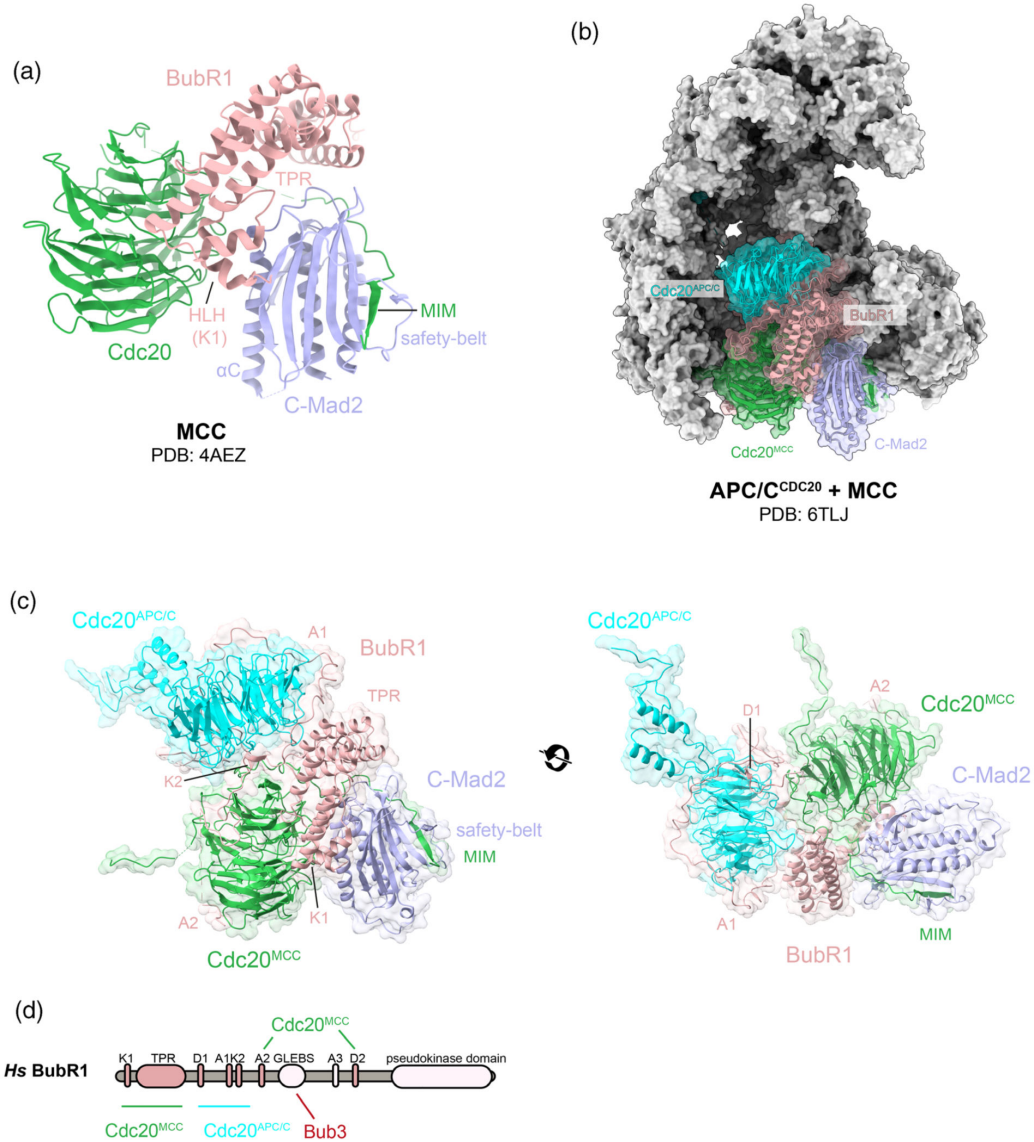
Structure of the mitotic checkpoint complex (MCC) and its inhibition of the anaphase-promoting complex/cyclosome (APC/C). (a) The crystal structure of the MCC from *S. pombe* (Protein Data Bank (PDB) 4AEZ).^14^ (b) A cryo-EM reconstruction of APC/C^MCC^ (PDB 6TLJ).^44^ The APC/C subunits are displayed using grey surface representation in ChimeraX,^198^ Cdc20^APC/C^ is colored in cyan, BubR1 in pink, Cdc20^MCC^ in green, and Mad2 in blue. (c) Two views of the MCC bound to and inhibiting Cdc20^APC/C^ (PDB 6TLJ).^44^ (d) Schematic representation of BubR1. BubR1 domains involved in interacting with either Cdc20^MCC^ or Cdc20^APC/C^ are shaded dark pink and are highlighted in the structure in (c). All structures are displayed using ChimeraX.^198^

A schematic of the key functional domains within BubR1, Bub3, Mad2, and Cdc20 is shown in Figure 2. Mad2 is a HORMA domain-containing protein that adopts two topologically distinct states: an “inactive” open state (O-Mad2) and an ‘active’ closed state (C-Mad2).^49–53^ Conversion of O-Mad2 into C-Mad2 involves a metamorphic rearrangement of its β-sheet (detailed in Section 3.1), which allows its C-terminal β-hairpin and its adjacent loop, termed the “safety-belt,” to entrap the Mad2-interacting motif (MIM) of Cdc20 to form the C-Mad2:Cdc20 complex (Figure 3a).^51,54^ The majority of Cdc20 is comprised of a WD40 domain which folds into a canonical seven-bladed β-propeller and binds to the tetra-tricopeptide repeat (TPR) domain, as well as the KEN1, ABBA2 (A2) and D-box2 (D2) motifs of BubRI at distinct positions on its β-propeller (Figure 3a).^14,55^ The N-terminal helix–loop (HLH) motif of BubR1 which contains the KEN1 box is sandwiched between the β-propeller of Cdc20 and the C-terminal α-helix of C-Mad2 (αC) (Figure 3a).^14^ Following the BubR1 HLH motif, the TPR domain makes further reinforcing contacts with Cdc20 and C-Mad2 (Figure 3a).^14^ Bub3 is made up of seven WD40 repeats that form a β-propeller arrangement and bind to the GLEBS motif of BubR1.^56,57^ Bub3 has never been seen in structures of the MCC suggesting it is only flexibly tethered to BubR1. Bub3 is not required for MCC association but has several important downstream functions discussed in Section 4.^17,58^ Bub3 is not part of the MCC in fission yeast.^59^

### 2.2 The molecular basis of MCC-mediated APC/C inhibition

The critical function of the MCC is to prevent the ubiqui-tination of cyclin B and securin by the APC/C when the APC/C is coactivated by Cdc20 (APC/C^Cdc20^).^13,24^ The APC/C is a 1.2 MDa cullin-RING E3 ubiquitin ligase, composed of 14 distinct proteins and 19 subunits (Figure 3b).^24,28,60–62^ The APC/C complex is stable throughout the entire cell cycle, but its activity is highly regulated. Temporal regulation occurs through two structurally related co-activators; Cdc20 and Cdh1, APC/C inhibitors, and reversible protein phosphorylation.^26,63,64^ APC/C coactivators, Cd20 and Cdh1, assist APC/C substrate recognition using their WD40 domains to identify KEN-boxes and D-boxes on substrates.^65–67^ The ABBA motif is additionally recognized by Cdc20 in humans and by Cdh1 in *S. cerevisiae*.^67,68^

The MCC is a potent APC/C^Cdc20^ inhibitor. It binds directly to APC/C^Cdc20^ and represses APC/C ubiquitination by blocking substrate recognition and by impairing the E3 ligase activity of the APC/C^24,25,27,28,31,69^ (reviewed in Ref.^24^). The MCC achieves this largely by two mechanisms: steric hindrance and inhibiting conformational change of the APC/C active site. The Cdc20 molecule which binds and co-activates APC/C (Cdc20^APC/C^), is a separate molecule from that of the Cdc20 incorporated into the MCC (Cdc20^MCC^) (Figure 3b,c).^70–73^ This gives Cdc20 the unique role of being both a critical activator and inhibitor of the APC/C. The MCC binds to the central cavity of APC/C^Cdc20^ by docking onto its substrate recognition module (Cdc20^APC/C^), forming an extensive interface to both APC/C and Cdc20^APC/C^ (Figure 3b).^24–28,31^ Cdc20^MCC^ and Cdc20^APC/C^ engage with each other through BubR1 which weaves between them (Figure 3c). BubR1 then uses a separate set of degrons (KEN2, A1, D1) from those used to interact with Cdc20^MCC^ (KEN1, A2, D2) to obstruct substrate recognition by Cdc20^APC/C^ (Figure 3a,c,d).^26–29^ Key to this is Mad2 which helps to position KEN1 of BubR1 to bind Cdc20^MCC^ (Figure 3c,d). The ability of the BubR1 degrons to act as pseudosubstrate inhibitors explains why these degrons are critical to MCC-mediated APC/C inhibition and robust checkpoint response.^74–78^ Additionally, the APC/C subunit Apc10 which contributes to Cdc20^APC/C^ substrate recognition, is separated from the substrate recognition site upon MCC binding to APC/C^Cdc20^. This is due to a conformational change within APC/C^Cdc20^ induced upon MCC binding which pushes Cdc20^APC/C^ away from Apc10 (Figure 3b). The TPR domain of BubR1 also binds to the winged-helix B (WHB) domain of APC2. This blocks UbcH10 (the E2 of APC/C) from binding the APC/C catalytic module and represses APC/C E3 ligase activity (Figure 3b).

## 3 The Kinetic Barrier To Cdc20:C-Mad2 Association

Rapid assembly of the MCC requires overcoming a kinetic barrier to C-Mad2:Cdc20 association.^20–23,79^ Our current understanding is that there are largely two impediments to their interaction: a naturally very slow conversion of O-Mad2 into C-Mad2 which is required to entrap the MIM of Cdc20,^20,53,79,80^ and repositioning of the Cdc20 MIM relative to O-Mad2 which is required to promote MIM accessibility and induce rapid Mad2 conversion.^5,46,48,49^ The former has been well studied while the latter only recently became recognized and is less well understood. The underlying mechanisms for both barriers will be presented and examined below.

### 3.1 The molecular details of Mad2 conversion

**Figure 4:**
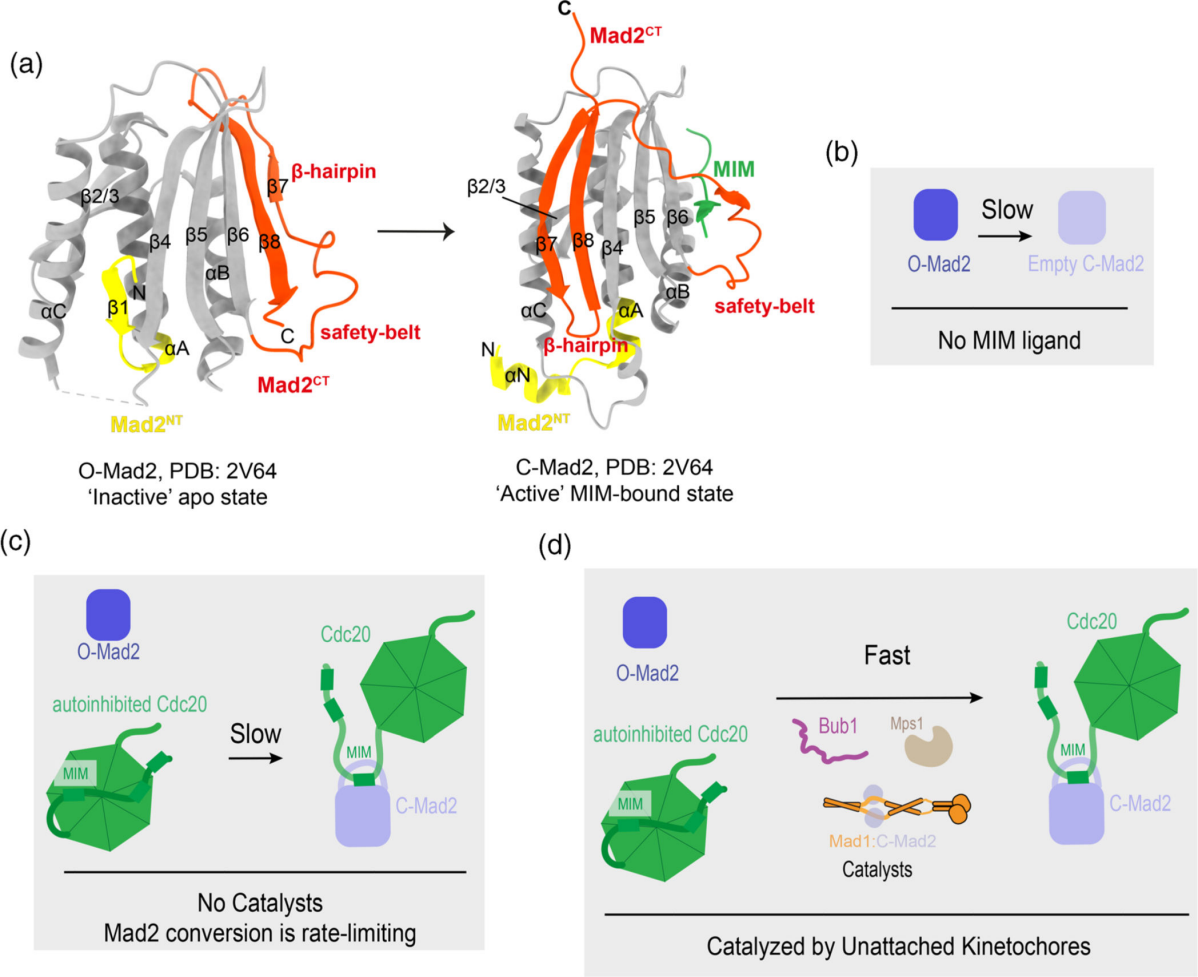
Mad2 conversion and the kinetic barrier to C-Mad2:Cdc20 association. (a) The conformational rearrangement of Mad2 during the O-Mad2 to C-Mad2 transition. On the left O-Mad2 is depicted, and on the right C-Mad2 with the Mad2-interacting motif (MIM) entrapped by the safety belt of Mad2 (PDB 2V64).^84^ The crystal structure was determined in the presence of a MIM peptide selected for its enhanced C-Mad2 affinity using phage display.^83^ The N- and C-terminal segments which undergo remodeling during Mad2 conversion are colored in yellow and red, respectively. (b-d) Schematics outlining relative rates at cellular concentrations of Mad2 conversion and C-Mad2: Cdc20 formation with or without ligand (Cdc20) and catalysts. (b) Conversion of O-Mad2 by itself into C-Mad2 is kinetically disfavored and thus conversion occurs very slowly. (c) Conversion of O-Mad2 and C-Mad2:Cdc20 association in the presence of full-length Cdc20 without accompanying catalysts is limited by the disfavoured and rate-limiting spontaneous conversion of Mad2 which is required to form C-Mad2: Cdc20. (d) Conversion of O-Mad2 and concomitant C-Mad2:Cdc20 formation is rapid in the presence of identified catalysts (Bub1, Mad1:C-Mad2, and Mps1 phosphorylation).

A variety of solution and crystal structures of Mad2 in the unbound and bound states have been determined to delineate the dramatic remodeling that Mad2 undergoes during the open-to-closed (O-to-C) Mad2 transition.^52,80–86^ Mad2 contains a central core (colored grey in Figure 4a), which includes a three-stranded anti-parallel β-sheet (β4-6) and three α-helices (αA-C) with a β-hairpin (β2-3) between the αA and αB helices (Figure 4a). The core of Mad2 is preserved during O-to-C Mad2 conversion while both the N- and C-terminus of Mad2 (colored yellow and red, respectively) undergo a metamorphic structural remodeling (Figure 4a). The disordered segment at the very C-terminus of O-Mad2, as well as the two adjacent β-strands (β7/8 colored red) which form a C-terminal β-hairpin, swing across the central face of Mad2, displacing the N-terminal β-strand in O-Mad2 (β1 colored yellow in O-Mad2) (Figure 4a). This ejected N-terminal β-strand then refolds into an α-helix (αN) and an extended αA-helix in C-Mad2 (colored yellow) (Figure 4a). The rearrangement of the C-terminal β-hairpin and its adjacent loop (the safety-belt), ultimately enables Mad2 to enclose the β-stranded Cdc20 MIM (colored green) (Figure 4a). This entrapment of the Cdc20 MIM during Mad2 O-to-C conversion has thus been coined the ‘safety-belt’ mechanism and results in a dramatic alteration of the secondary structure and tertiary structure of Mad2.^85^ The existence of transient intermediate(s) of Mad2 (often referred to as I-Mad2) which occur during Mad2 conversion have been suggested, however, the existence and/or relative importance of these intermediate states has yet to be fully examined.^81,87^

### 3.2 Mad2 conversion is kinetically disfavored

In cells, the SAC response is established within minutes of kinetochore-microtubule detachment.^65,88^ In vitro, however, formation of the MCC from O-Mad2, BubR1: Bub3, and Cdc20 at physiological concentrations is extremely slow, with the half-life being about 220 mins and requiring overnight incubation to reach equilibrium (Figure 4b,c).^23^ This is because the topological rearrangement which occurs during the O-to-C conversion of Mad2 requires large activation energy (Figure 4a).^20^ This means that spontaneous O-to-C conversion of Mad2, which is required to entrap the MIM of Cdc20 to form C-Mad2:Cdc20, is kinetically disfavored (naturally very slow) and rate-limiting for MCC formation.^20–22^ As detailed below, subsequent studies focused on discovering the molecular mechanisms responsible for overcoming this rate-limiting barrier.

### 3.3 C-Mad2:Cdc20 formation occurs on the Mad1:C-Mad2 platform

**Figure 5:**
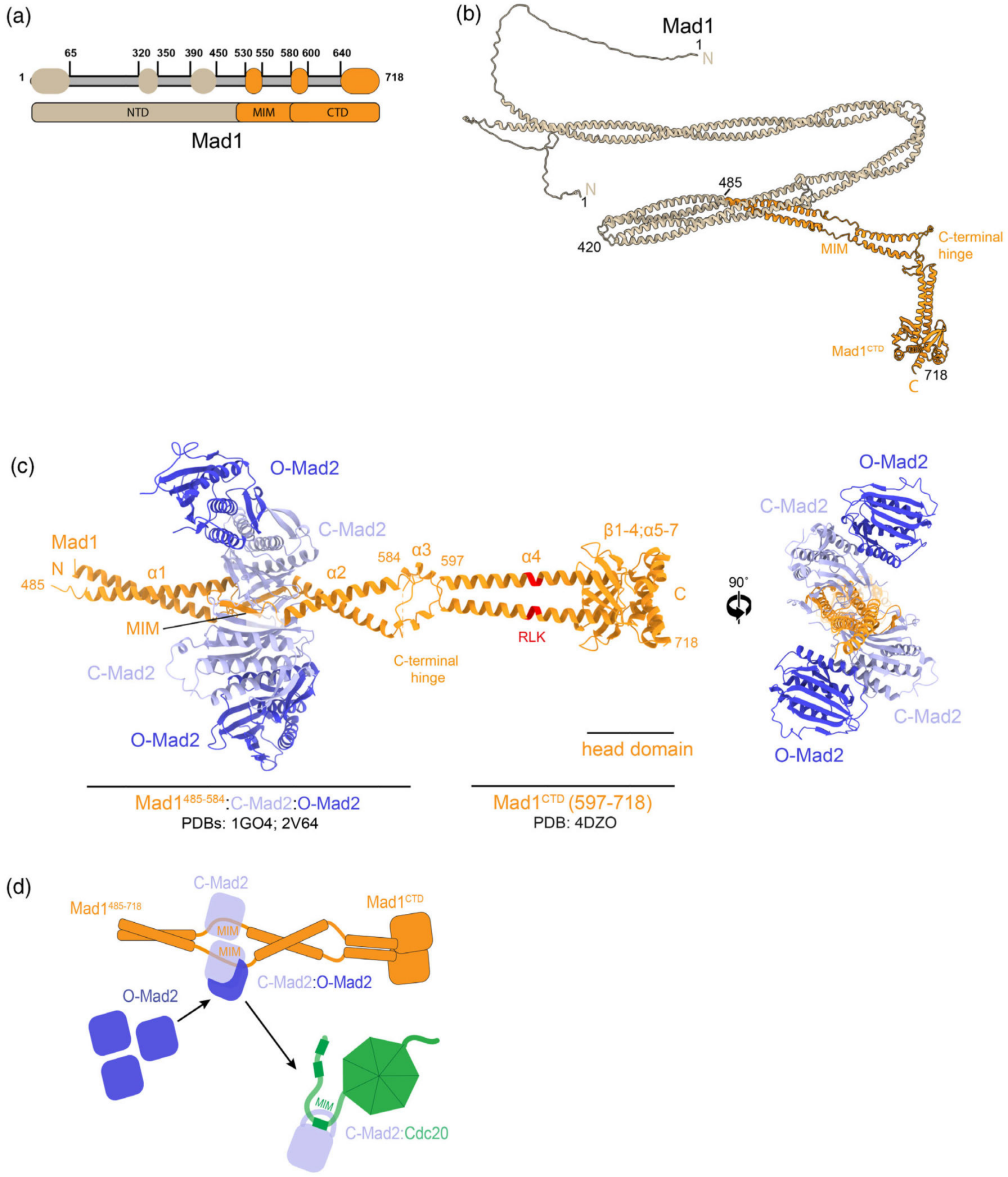
Mad1:C-Mad2 is the platform for Mad2 conversion. (a) A schematic diagram of Mad1 where non-coiled-coil segments are depicted as ovals as predicted by COILS.^199^ (b) An AlphaFold2 prediction of full-length dimeric Mad1, where residues 1−485 and 486−718 are colored brown and orange respectively.^93,94^ (c) Side and bottom views of Mad1^485−718^:C-Mad2:O-Mad2 hexamer. The Mad1 dimer encompassing residues 485−584 is depicted in orange, bound to two C-Mad2 molecules in light blue (PDB 1GO4).^85^ Two O-Mad2 molecules (dark blue) dimerized to C-Mad2 are fitted using the structure of the O:C Mad2 dimer (PDB 2V64).^84^ The coiled-coil is then interrupted by a flexible 16-residue segment, termed the C-terminal hinge, which is modelled by AlphaFold2 to contain a novel short α-helix.^93,94^ The Mad1 C-terminal hinge facilitates fold-over of the Mad1 C-terminus (Mad1^CTD^; residues 597−718), which comprises a coiled-coil stem ending in a globular head domain featuring an RING, WD40, DEAD (RWD) fold (PDB 4DZO).^45,153^ The RLK motif within Mad1^CTD^ is highlighted in red. (d) The Mad1:C-Mad2 platform targets O-Mad2 to unattached kinetochores. O-Mad2 then undergoes conversion to C-Mad2, releasing it from Mad1:C-Mad2 and simultaneously binding the Mad2-interacting motif (MIM) of Cdc20 positioned nearby.

One of the first proteins identified as essential to the checkpoint response is the highly evolutionarily conserved protein Mad1.^89–91^ Mad1 is an elongated protein that self-dimerizes through a series of coiled-coil α-helices (Figures 2 and 5a,b).^90–92^ Currently, there is no experimentally determined structure of full-length Mad1 or its N-terminal 485 residues. AlphaFold2^93,94^ generates a model of the N-terminus of Mad1 which largely consists of long coiled coils that possibly fold onto themselves (Figure 5b).^93,94^ In contrast the C-terminus of Mad1 (Mad1^485–718^) is well studied. The coiled-coil formed from residues 485–584 is interrupted by a MIM, spanning residues 530–550, which entraps one molecule of Mad2 per subunit to make the Mad1:C-Mad2 tetramer (Figure 5a-c).^85^ Each Mad1-bound C-Mad2 can then target and self-dimerize to cytosolic O-Mad2, to make a Mad1:C-Mad2:O-Mad2 pentamer or hexamer (Figure 5c).^54,85,86,95,96^ It is the O-Mad2 molecule that is asymmetrically dimerized to Mad1-bound C-Mad2 which then undergoes conversion to C-Mad2 to generate the C-Mad2:Cdc20 complex (Figure 5d).^85,92,95,97,98^

Several experiments have been key to unveiling this mechanism (first coined the “Mad2 template model”^95^), by which C-Mad2:Cdc20 formation requires Mad1:C-Mad2 as the platform for O-to-C Mad2 conversion. The SAC cannot be triggered in cells where Mad2 mutants locked in either the open or closed state are targeted to kinetochores, or by targeting a Mad2 R133A mutant which is unable to self-dimerize.^95,99,100^ Mad1 overexpression will titrate away free O-Mad2 and abrogate the checkpoint.^97^ P31^comet^, an adaptor protein important for MCC disassembly, which is homologous to O-Mad2 and can bind the Mad1:C-Mad2 complex, blocks O-Mad2 binding and prevents MCC formation.^40,86,101–104^ Checkpoint dysfunction occurs if a Mad1 MIM mutant (K541A/L543A), which cannot bind Mad2 is used.^105^

Originally it was proposed that on the Mad1:C-Mad2 platform O-Mad2 first converts into a ligand-free “empty” C-Mad2 which can then rapidly associate with Cdc20.^52,54^ However, at cellular concentrations Cdc20 does not bind to C-Mad2 at an appreciable rate.^46^ Binding of C-Mad2 to Cdc20 at cellular concentrations only occurs when the MIM of Cdc20 is presented as a peptide (Cdc20^MIM^) or when the N-terminus preceding the MIM of Cdc20 is truncated (Cdc20^111-C^).^46^ This is likely because in either scenario the MIM can thread under the already remodeled safety-belt, whereas this is prevented in full-length Cdc20. This is also supported by experiments that show that the checkpoint is abrogated in cells where C-Mad2 is the only available Mad2 species.^99,106,107^ Additionally, because C-Mad2 cannot dimerize with C-Mad2 already bound to Mad1 (although an unliganded C-Mad2:C-Mad2 dimer has been shown to exist^82^), the Mad1:C-Mad2 platform cannot be utilized when only C-Mad2 is present.^95^ Altogether this indicates that C-Mad2: Cdc20 formation occurs on the Mad1:C-Mad2 platform, where O-to-C Mad2 conversion is both necessary and simultaneous with Mad2 safety-belt entrapment of Cdc20 (Figure 5d). How the kinetic barrier to Mad2 conversion affects Mad1:C-Mad2 formation in cells is an interesting question. Our current understanding suggests that there is no intricate mechanism required to assemble Mad1:C-Mad2. This may be because Mad1 and Mad2 have ample time to associate under non-catalytic conditions, or it may be that the MIM of Mad1 can spontaneously thread under the safety-belt of C-Mad2, as compared to Cdc20 where this pathway is not available.

### 3.4 A Bub1:Mad1:C-Mad2 scaffold catalyzes C-Mad2:Cdc20 formation

Dimerization of O-Mad2 to the Mad1:C-Mad2 platform has been shown to directly accelerate the O-to-C Mad2 transition.^20,23,46,79^ Therefore, Mad1-bound C-Mad2, not only acts as a platform for recruitment and conversion of O-Mad2 but also acts directly to encourage Mad2 conversion.^84,85,96,108^ How Mad2 dimerization directly promotes O-to-C Mad2 remodeling remains unclear. One hypothesis is that O-Mad2 dimerized to Mad1-bound C-Mad2 is in a partially “activated” state, whereby the C-terminal β7/8-hairpin of O-Mad2 which must become detached during the structural metamorphosis into C-Mad2, is somehow more susceptible to spontaneous detachment (Figure 4a).^81,108^ However, the interaction of O-Mad2 with Mad1:C-Mad2, albeit essential for checkpoint function, promotes only a minor acceleration of MCC assembly, indicating that other components are required for full catalytic acceleration.^23,46,88^

An elegant FRET system that directly measures O-Mad2 conversion and concomitant Cdc20:C-Mad2 formation by assessing CFP-Cdc20 binding to Mad2 conjugated to a TAMRA fluorophore has been established.^23,46,109^ This assay has identified and quantified other factors which directly contribute to catalyzing C-Mad2:Cdc20 formation in vitro. An MCC-mediated APC/C inhibition substrate ubiquitination assay also contributed to these findings.^47^ These assays identified that in addition to Mad2 dimerization, Mad1:C-Mad2, Cdc20, phosphorylated Bub1, and phosphorylation of the Mad1 C-terminus are all key contributors to catalytic C-Mad2:Cdc20 association.^23,46,47^ The O-to-C transition of Mad2 and simultaneous binding to Cdc20 in the presence of identified catalysts (Mad1:C-Mad2, Bub1, and Mps1 phosphorylation—“the catalytic scaffold”) in vitro, accelerates O-Mad2 binding to Cdc20 to within minutes.^23,46^ This is a 35-fold increase which is near physiologically relevant rates (Figure 4d).^23,46^ It is important to highlight that as these assays are conducted in vitro, the catalytic contribution of Bub1 and Mad1 identified is complementary to their roles in recruiting checkpoint proteins to kinetochores (discussed in Section 4). This is confirmed by the importance of Bub1 in SAC signaling even when Mad1 is artificially tethered to kinetochores.^110,111^ Interestingly, the N-terminus of Mad1 is not required for catalysis in vitro, although a Mad1 truncation encompassing residues 420–718 was found to be a slightly stronger catalyst than the traditionally used Mad1^485–718^ (Figure 5a).^23^

### 3.5 The catalytic scaffold functions to present the Cdc20 MIM to O-Mad2

Understanding how the scaffold functions to accelerate Mad2 conversion and simultaneous Cdc20 binding is of key interest. Spontaneous O-to-C conversion of Mad2 can be induced by presenting O-Mad2 with a peptide encompassing the MIM of either of its ligands (Mad1^MIM^ and Cdc20^MIM^).^49,80,83^ The molecular mechanisms for how the MIM directly induces Mad2 conversion remain a fundamental question. One hypothesis is that the MIM transiently binds O-Mad2, and either directly or allosterically triggers detachment of the β7/8-hairpin of Mad2, making it available to entrap the MIM (Figure 4a). It may also be that this β7/8-hairpin of O-Mad2 is in a more “breathable” intermediate state than the experimentally determined O-Mad2 structures suggest. This could allow the MIM to instantaneously trigger its own entrapment upon spontaneous β7/8-hairpin detachment and stabilize the C-Mad2 state. Further clarifying these hypotheses is key to completing our understanding of how the catalytic scaffold directly catalyzes C-Mad2:Cdc20 formation but remains a challenge due to the fleeting nature of these states.

The ability of the MIM to induce O-to-C Mad2 conversion does not however explain how rapid Mad2 conversion is achieved at unattached kinetochores. This is because even at cellular concentrations Cdc20^MIM^ does not have a fast association rate with O-Mad2.^46^ At physiological concentrations Cdc20^MIM^ and full-length Cdc20 associate with O-Mad2 at comparably slow rates.^46^ The ability of Cdc20^MIM^ to induce Mad2 conversion in a concentration-dependent manner does however imply that the critical function of the catalytic scaffold in cells is to position the MIM of Cdc20 close to O-Mad2 to trigger their rapid association.^49^ These in vitro MCC assembly assays also identify that the catalytic scaffold accelerates full-length Cdc20 binding to O-Mad2 significantly, while binding to Cdc20^MIM^ is accelerated only mildly.^46^ This suggests that the catalytic scaffold acts directly on Cdc20 to locally concentrate and spatially constrain the MIM close to O-Mad2 to trigger Mad2 conversion. In support of this, even when tested in vitro, preventing O-Mad2 dimerization to Mad1:C-Mad2 or preventing Cdc20 association to the scaffold by eliminating its interaction with Mad1 and Bub1 (discussed in Section 6) abolishes the ability of the catalytic scaffold to facilitate catalytic C-Mad2:Cdc20 formation.^46^

## 4 Assembly Of The Catalytic Scaffold By Unattached Kinetochores

### 4.1 The master regulator: Mps1

SAC activation and error correction is a highly regulated signal transduction cascade under the surveillance of several kinases, including Aurora B, Cdk1, and Mps1, as well as the phosphatases, PP1 and PP2A.^112–114^ At the apex of SAC signaling is the evolutionarily conserved kinase Mps1 (monopolar spindle 1/TTK). Mps1 is responsible for sequentially recruiting SAC components onto the outer kinetochore, facilitated by the KMN (Knl1, Mis12 and Ndc80) network.^115–119^ Ultimately this is how the SAC assembles the catalytic scaffold to rapidly generate the MCC.^5^ Mps1 also releases the Mad1:C-Mad2 complex from nuclear pores during mitotic entry,^120,121^ and phosphorylates Mad1 to promote catalytic C-Mad2:Cdc20 formation.^46,47,49^ Overexpression of Mps1 can activate the checkpoint even in the presence of intact kinetochore-microtubule attachments, a mechanism which occurs through hyperphosphorylation of Bub1 and Mad1.^118^ Mps1 persistence at kinetochores, such as through KMN tethering, delays anaphase onset and can induce cell death.^122^ Furthermore, inhibiting Mps1 by adding reversine, a selective inhibitor of Mps1 kinase activity, impedes the localization of nearly all downstream checkpoint proteins and creates a defective checkpoint.^122–124^

Despite extensive studies on Mps1, the mechanism of its kinetochore localization remains poorly understood. The prevailing model suggests that the Ndc80 complex of the KMN network is the direct receptor of Mps1 at kinetochores.^125–128^ The Ndc80 complex is composed of Ndc80 (Hec1 in humans), Spc24, Spc25, and Nuf28.^129–131^ The N-terminal calponin-homology (CH) domain of Ndc80 binds microtubules directly.^8,11,132^ Mps1 contains an N-terminal TPR domain and an N-terminal extension (NTE) (Figure 2). The TPR domain and NTE self-interact prior to mitosis, but during mitosis this autoinhibition is relieved and both bind to the CH domain of Ndc80 to recruit Mps1, with the NTE being the critical interaction site for Ndc80.^126,127,133–135^ Consistent with these results, deletion of the Mps1 N-terminus abolishes Mps1 kinetochore targeting and SAC signaling.^125^

This binding site of Mps1 to the CH domain of Ndc80 is at least partially mutually exclusive with the microtubule-binding site on Ndc80.^134,135^ This has led to the suggestion that Mps1 localization is a direct sensor for microtubule attachment and plays a key regulatory role in initiating the Mps1 phosphorylation-dependent signaling cascade to assemble the MCC.^5,136^ However, Mps1 was found to localize to microtubule-attached kinetochores and facilitate microtubule release during Aurora B-dependent error correction.^137^ This suggests Mps1 kinetochore localization is not only limited to recruitment by microtubule-free Ndc80, and further work will be required to clarify these pathways. Aurora B also plays a role in Mps1 localization.^112,138,139^ Inhibition of Aurora B prevents Mps1 recruitment, while tethering Mps1 bypasses the SAC assembly requirement for Aurora B.^139^ Aurora B might contribute to Mps1 kinetochore targeting by phosphorylating the Ndc80 CH domain which then enhances Mps1 binding.^140^ Aurora B activity also helps alleviate the inhibitory effect of the Mps1 TPR:NTE interaction.^125^ Another possibility is that Mps1 self-phosphorylates its NTE, to relieve autoinhibition.^112,125,133^

### 4.2 Mps1 phosphorylation sequentially assembles the catalytic scaffold for MCC formation

**Figure 6:**
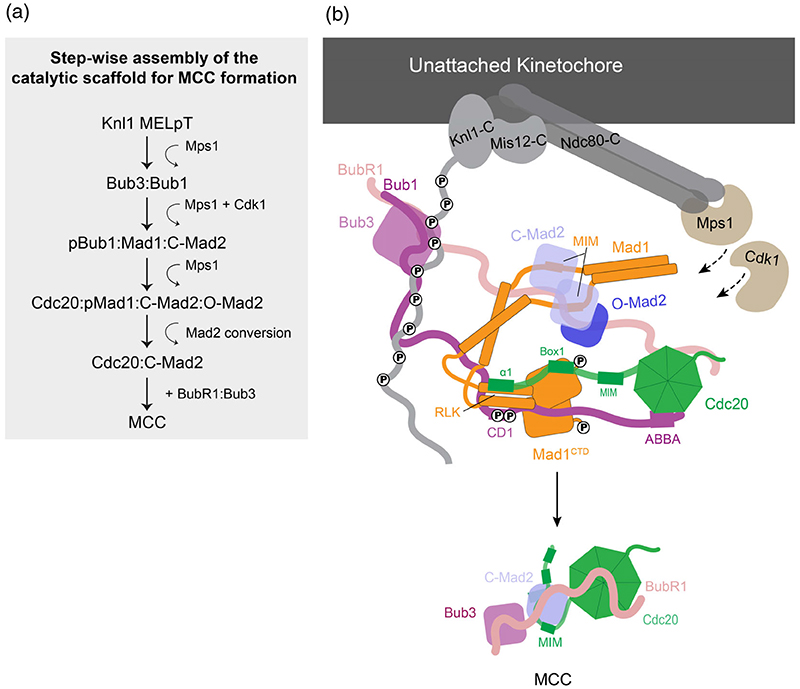
Mps1 phosphorylation-dependent stepwise assembly of the catalytic scaffold for MCC formation. (a) The sequential steps of catalytic MCC assembly onto unattached kinetochores are outlined. (b) A schematic of the catalytic scaffold assembled onto the outer kinetochore. Phosphorylated Knl1 Met-Glu-Leu-Thr (MELT) motifs recruit the Bub3:Bub1 complex, which is sequentially phosphorylated by Cdk1 and Mps1 on the Bub1 CD1 domain. Phosphorylated Bub1 CD1 recruits the Mad1:C-Mad2 complex and O-Mad2 is recruited through self-dimerization with Mad1-bound C-Mad2. Mad1 is further phosphorylated by Mps1 which promotes interaction with the N-terminus of Cdc20. The catalytic scaffold then functions to bring the Mad2-interacting motif (MIM) of Cdc20 near O-Mad2 to promote near instantaneous conversion of O-Mad2 into C-Mad2, releasing it from Mad1:C-Mad2 and simultaneous entrapment of the Cdc20 MIM by the C-Mad2 safety-belt. C-Mad2:Cdc20 then binds BubR1:Bub3 to generate the MCC.

Stepwise assembly of the catalytic scaffold by Mps1 is outlined in Figure 6. Mps1 phosphorylates several MELT (Met-Glu-Leu-Thr) motifs on the outer kinetochore subunit Knl1.^141–147^ These phosphorylated MELTs recruit Bub3, which is already bound to Bub1 of the Bub1:BubR1 dimer.^145,146,148–150^ Mps1 then phosphorylates the conserved domain 1 (CD1) of Bub1 at Thr461, after being primed by Cdk1 phosphorylation at Ser459.^47,142,151,152^ This enables phosphorylated Bub1 CD1 to bind and recruit the Mad1:C-Mad2 complex through an interaction with an RLK (Arg-Leu-Lys) motif within the C-terminal domain of Mad1 (Mad1^CTD^).^45,47,151–153^ Mps1 also phosphorylates Mad1^CTD^ to promote an interaction with the N-terminus of Cdc20, and this together with Bub1-mediated Cdc20 kinetochore recruitment, assembles the catalytic scaffold for C-Mad2:Cdc20 formation.^46,47,49^

### 4.3 Bub1 and BubR1 kinetochore targeting

#### 4.3.1 Knl1 MELT motifs

**Figure 7:**
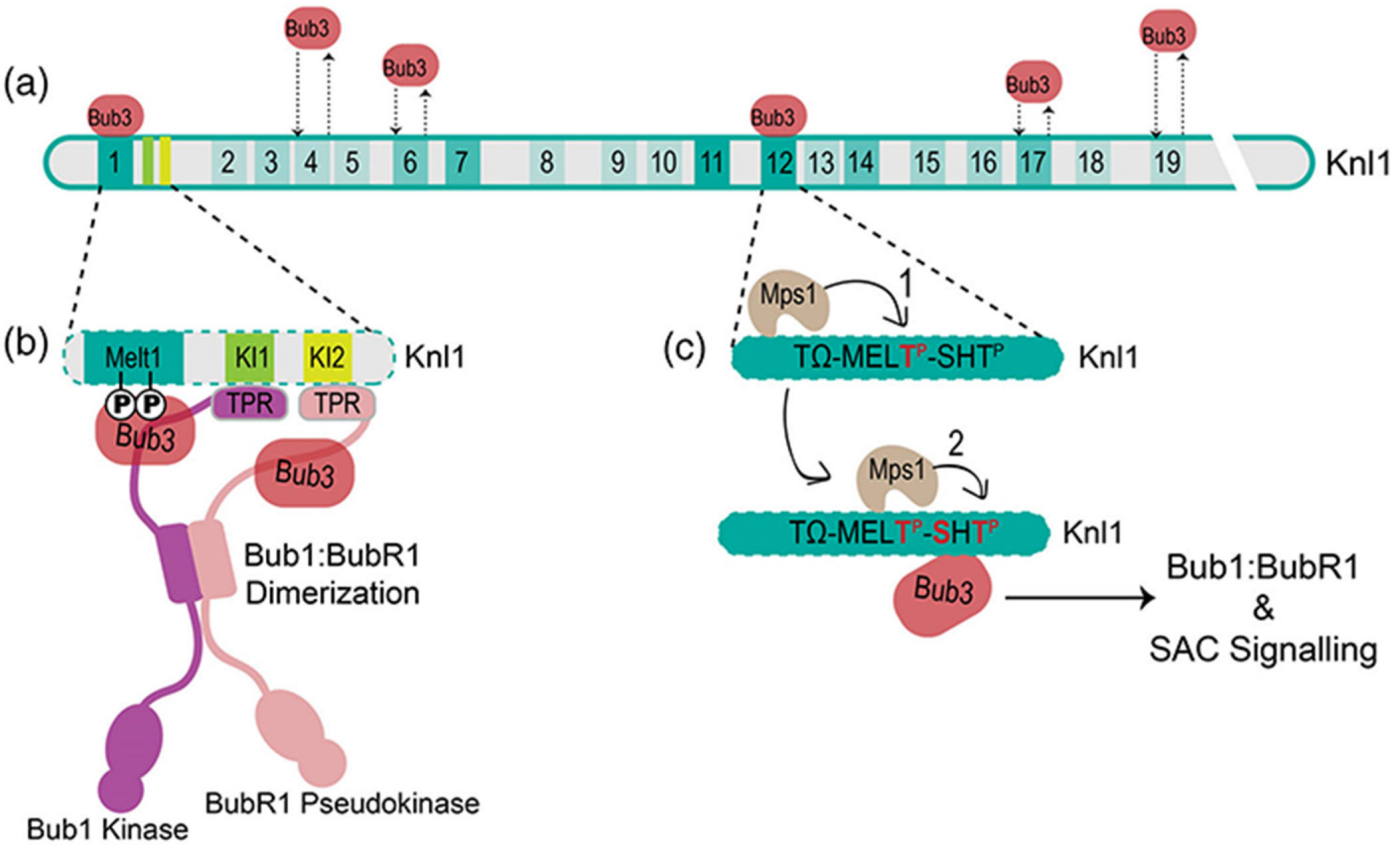
Knl1 MELTs and the KI1 and KI2 motifs in SAC signaling. (a) A schematic depiction of Knl1 with its 19 Met-Glu-Leu-Thr (MELT) repeats. The activity of each MELT, as defined by the ability of the MELT to recruit the Bub3:Bub1 complex for SAC signaling, corresponds to a color gradient (the darker the more active).^145,146^ The strength of the Bub3:MELT interaction is also depicted by the arrow length (the shorter the stronger the binding). (b) A close-up of the N-terminus of Knl1 where MELT1 is immediately proceeded by the KI1 and KI2 motifs which bind to the tetratricopeptide repeat (TPR) lobes of Bub1 and BubR1, respectively. This mechanism enhances Bub1/BubR1 recruitment and is unique to some metazoans including humans. (c) Strong MELT motif activity requires sequential multisite phosphorylation of the Knl1:Bub3 interface by Mps1. Mps1 first phosphorylates the threonine MELT residue (MELpT) which enhances Mps1 phosphorylation at the SHT motif. Strong recruiters of Bub3:Bub1 require the SHT motif to enhance the Bub3:MELT interaction.

Knl1 is phosphorylated by Mps1 at its conserved MELT motifs ([M/I/L/V]-[E/D]-[M/I/L/V]-pT) which then act as a recruitment platform for Bub3:Bub1 and possibly BubR1:Bub3 (Figures 2 and 7a).^143,149,150,154^ Binding occurs through the methionine and phosphorylated threonine of the MELpT sequence which dock onto the hydrophobic and basic patches of Bub3, respectively (Figure 7b).^150^ Interestingly the number of MELT copy numbers within Knl1 varies substantially across organisms, from 2 to 27 MELT repeats, suggesting that Knl1 is a rapidly evolving protein.^145,147,155^ Human Knl1 has 19 MELT-like motifs (Figure 2) as compared to *S. pombe* (Spc7) which has 8, and *S. cerevisiae* (Spc105) which only has 5. Not all MELT motifs have the same activity towards Bub3 recruitment (Figure 7a).^146^

MELT motifs also have other species-specific motifs adjacent to them which likely alter the affinity of Knl1 for Bub3:Bub1. In humans, 10 MELTs have an N-terminal TxxΩ motif (x, any amino acid; Ω, aromatic) which is critical for Bub1 recruitment by an unknown mechanism (Figure 7c).^145^ The most active MELTs also have an indispensable Ser-His-Thr (SHT) motif C-terminal to the MELT (Figure 7c).^146^ MELT motifs first recruit Mps1 after which MELT phosphorylation primes SHT motif phosphorylation and Bub3 recruitment, a mechanism thought to be unique to vertebrate Knl1.^146^ Furthermore, in vertebrate orthologs, the N-terminal MELT1 has two 12-residue upstream KI motifs which can recruit Bub1 and BubR1 TPR lobes (Figure 7b).^149,156–159^ However, mutations in the KI motifs do not abolish Bub1/BubR1 kinetochore localization and are not required for the checkpoint.^143,159^ Detailed analyses have shown that KI motifs are MELT enhancers, making MELT1 the strongest recruiter of Bub1:Bub3 and the only MELT which can sustain the SAC by itself.^159^ Overall, the abundance of MELTs and the difference in their affinities suggest a mechanism by which eukaryotic cells can control the strength of SAC signaling in various scenarios of segregation errors.^146,160^

#### 4.3.2 Bub1:BubR1 dimerization

Bub1 and BubR1 evolved from several duplication events with a common ancestral gene.^147^ They share similar domains, including TPR, GLEBS, KEN, and ABBA motifs (Figure 2), and yet have very different roles in SAC signaling.^149^ Both Bub1 and BubR1 form stable mutually exclusive complexes with Bub3, both using the GLEBS domain.^57,161^ Initially, it was thought that both Bub1 and BubR1 were recruited to Knl1 through Bub3. However, Bub3 is not essential for BubR1 recruitment and BubR1 does not stabilize the Bub3:Knl1 interaction as Bub1 does.^149,150^ This is because Bub1 contains a loop upstream of the GLEBS Bub3 binding domain which enhances Bub3 binding to phosphorylated MELTs on Knl1.^149^ Interestingly the same loop in BubR1 is required for enhancing the ability of the MCC to inhibit APC/C^Cdc20^.^58^ Subsequently, it was discovered that kinetochore localization of BubR1 relies on heterodimerization of Bub1 and BubR1 through a novel domain within their respective GLEBS domains, termed the Bub1-BubR1 dimerization (BBD) domain (Figures 2 and 7b).^149^ Heterodimerization of Bub1:BubR1 is conserved in fission yeast where dimerization occurs through their tetratricopeptide repeat (TPR) domains.^162^ However, it has also been suggested that the pool of BubR1 dimerized to Bub1 is required for BubR1-mediated chromosome alignment.^16^ While the pool of BubR1 required for SAC signaling interacts directly with phosphorylated MELT motifs through Bub3.^16^ Therefore, the exact pathways of BubR1 kinetochore targeting remain debated.

#### 4.4 Mad1 targeting

Mad1 is targeted to kinetochores as a constitutive heterotetrameric Mad1:C-Mad2 complex.^85,92,95,97,163^ Kinetochore targeting of Mad1:C-Mad2 is crucial to SAC signaling. Mad1:C-Mad2 is the kinetochore receptor for the O-Mad2 molecule which undergoes conversion into C-Mad2 and is incorporated into the MCC.^85,95–98^ Additionally, the C-terminal domain of Mad1 plays a critical role in presenting Cdc20 MIM to O-Mad2 during catalytic C-Mad2:Cdc20 formation (discussed in Section 6).^5,46,47,49,91^ Artificially tethering Mad1 to kineto-chores can maintain an active checkpoint even after all kinetochores are attached.^164^ Additionally, reactivation of the checkpoint after SAC silencing can be achieved by using an FRB/FKBP rapamycin system to conditionally target Mad1 to kinetochores.^165^ Several studies have also shown that the strength of the SAC response is dictated by Mad1:C-Mad2 kinetochore levels.^88,160^

##### 4.4.1 Bub1 is the principal Mad1 receptor for MCC formation

In both humans and yeast Bub1 is the primary kinetochore receptor of Mad1.^5,136^ Mps1 phosphorylation of a central conserved domain 1 (CD1) of Bub1, recruits the C-terminus of Mad1 through direct interaction with the RLK motif (Figures 2 and 6b).^45,47,151,152,166^ In budding yeast, two phosphorylation sites within Bub1 CD1 are important for Mad1 recruitment, Thr453 and Thr455, with Thr455 being the primary contributor.^47,152^ In humans, the equivalent two sites (Ser459 and Thr461) are required for Mad1 kinetochore targeting.^47,152^ Just as for budding yeast only the second site (Thr461) is critical to the human Bub1-Mad1 interaction in vitro. It was subsequently discovered that in humans these two sites are sequentially phosphorylated. Cdk1 first phosphorylates Ser459, which then primes Mps1 phosphorylation of Thr461.^45,47,152^ This mechanism of priming is confirmed in the crystal structure of human Bub1^CD1^:Mad1^CTD^ where the phosphate of pThr461 of Bub1^CD1^ directly contacts Arg617 of the Mad1 RLK motif, whereas Bub1 pSer459 is part of the flexible N-terminus of Bub1^CD1^ and does not contact Mad1.^45^

##### 4.4.2 Is Rod:Zw10:Zwilch a direct receptor for Mad1:C-Mad2?

Contrary to yeast where Bub1 is the only kinetochore receptor of Mad1, it has been proposed that in humans other pathways exist for Mad1 kinetochore localization. One of the main contenders is the metazoan-specific Rod:Zw10:Zwilch (RZZ) complex.^110,167–174^ The RZZ complex comprises a fibrous biopolymer that forms the corona of the kinetochore, an expansion process that promotes microtubule capture and SAC signaling.^175,176^ RZZ and its dynein-adaptor Spindly also recruit the major retrograde motor dynein-dynactin to coordinate chromosome congression, corona disassembly (also referred to as corona “stripping” or “shedding”), and SAC silencing.^5,177–180^ Key to this process is the removal of Mad1:C-Mad2 which effectively halts MCC formation.^5,35,171,175^

Although the role RZZ plays in the removal of Mad1: C-Mad2 from kinetochores seems quite clear, the specific contribution RZZ has on Mad1:C-Mad2 kinetochore recruitment remains highly debated.^5,110,136,171–174,181^ A direct interaction between Mad1 and RZZ has yet to be detected, although Mad1 has been found in RZZ immunoprecipitants.^170,171,182,183^ Recently, it has been shown that corona-localized cyclin B1 directly binds the N-terminus of Mad1.^184^ This may suggest that the Cdk1-cyclin B1 complex tethers Mad1 to the corona, and thus RZZ may only indirectly contribute to Mad1 kinetochore localization. Although only the C-terminal 485–718 residues of Mad1 are required for in vitro MCC assembly,^23^ the rest of Mad1 contains a long N-terminal coiled-coil that likely spans over 80 nm in length (Figure 5a,b). The interaction of Mad1 to cyclin B1 localized at the outer corona might therefore directly scaffold the SAC.^184,185^

Ultimately, the specific role other pathways play in Mad1 kinetochore recruitment for MCC assembly remains elusive and further investigation is required. It will be particularly difficult to determine if these pathways are independent or synergistic with Bub1-mediated Mad1 kinetochore recruitment. Clarifying pathways to Mad1 kinetochore recruitment has been complicated by recent reports that Bub1 is not essential to SAC signaling and that Mad1:C-Mad2 recruitment does not require Bub1.^173,186,187^ However, further efforts clarified that residual Bub1 from incomplete depletions using RNAi or CRISPR-Cas9 was sufficient to catalyze MCC formation and support SAC signaling.^110,188,189^ Because Bub1 (and the Mad1:C-Mad2 it recruits) work catalytically in MCC assembly, incomplete depletion may remain compatible with checkpoint function under many conditions.

### 4.5 Cdc20 targeting

In *C. elegans*, Cdc20 recruitment is solely dependent upon the ABBA motif of Bub1.^190^ In humans, the exact recruitment pathway of Cdc20 remains unclear. Bub1 and BubR1 contain multiple different Cdc20 binding motifs, several of which are required for SAC signaling and contribute to Cdc20 kinetochore localization.^67,148,152,191,192^ It seems likely that the Bub1 ABBA motif, which immediately precedes its KEN1 motif (Figure 2), is the critical kinetochore receptor of the specific pool of Cdc20 for MCC formation, after which it facilitates transfer to BubR1 during catalytic MCC formation.^5,67,152,181^ Removal of the Bub1 ABBA motif has a dramatic effect on SAC signaling, whereas removing KEN1/2 and the Bub1 kinase domain does not.^67,152,181^ The KEN1 motif of Bub1 is the predominant recognition site for Bub1 ubiquitin-mediated degradation by the APC/C.^193^ KEN2, which is directly N-terminal to the start of the kinase domain of Bub1, mainly functions in the recruitment of Cdc20 for Bub1-mediated phosphorylation.^194–196^ Ultimately, clarifying the kinetochore recruitment pathways of Cdc20, as well as BubR1 and Mad1 remains an outstanding challenge.

## 5 Molecular Architecture Of The Catalytic Scaffold

**Figure 8:**
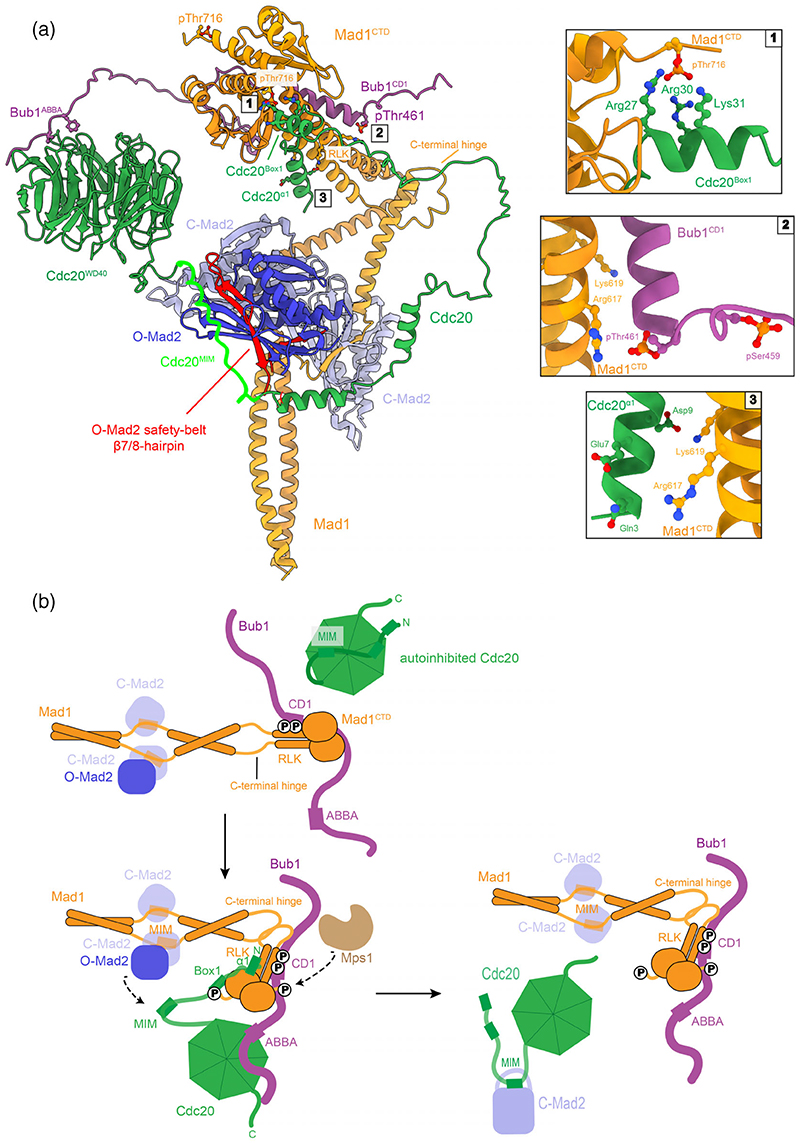
The molecular basis for catalytic C-Mad2:Cdc20 formation by the Bub1:Mad1 scaffold. (a) Model of pBub1:Cdc20:pMad1:C-Mad2:O-Mad2 complex (the mitotic checkpoint complex [MCC]-assembly scaffold) posed for catalytic C-Mad2:Cdc20 formation.^49^ The model was generated using PDBs 1GO4, 2V64, 6TLJ, 7B1F,^44,45,84,85^ and the AlphaFold2^93,94^ models of Bub1^448–534^:Mad1^CTD^:Cdc20 and the folded model of Mad1^485–718^ and are displayed using ChimeraX. Residues for the Mad1 pThr716 and Cdc20 Box1, Cdc20 α1 Asp9 and Mad1 Lys619, Mad1 Arg617 and Bub1 pThr461 interactions are depicted as sticks, as well as the Bub1 ABBA interaction with the Cdc20 WD40 domain. Key side-chain interactions are highlighted in the three boxes to the right of the model. (b) A schematic of catalytic MCC formation by the MCC-assembly scaffold. The doubly phosphorylated Bub1 CD1 domain targets the Mad1:C-Mad2 complex to kinetochores. Phosphorylation of the C-terminus of Mad1 at Thr716 promotes its interaction with the N-terminus of Cdc20. Interaction between the WD40 domain of Cdc20 and the ABBA/KEN1 motif of Bub1 also occurs and this likely promotes Cdc20 kinetochore targeting and positions Cdc20 close to Mad1:C-Mad2. The tripartite Bub1:Mad1:Cdc20 interaction together with Mad1^CTD^ fold-over then promotes Cdc20 Mad2-interacting motif (MIM) accessibility and repositioning close to O-Mad2 to trigger spontaneous C-Mad2:Cdc20 association. The Cdc20:C-Mad2 complex then rapidly binds BubR1:Bub3 to generate the MCC.

The catalytic scaffold for Cdc20:C-Mad2 formation is formed from Bub1 and Mad1:C-Mad2. Recent advances have provided the first structural model of the entire assembly, which allows visualizing how the catalytic scaffold would poise Cdc20 and Mad2 for their association (Figure 8).^49^

### 5.1 The Mad1:C-Mad2 platform

Tetrameric Mad1:C-Mad2 forms the base of the catalytic scaffold (Figure 8a). The coiled-coil of Mad1^485–718^ is first interrupted by the MIM (residues 530–550) which entraps one molecule of C-Mad2 per subunit (Figures 5c and 8a).^85^ O-Mad2 can then self-dimerize with Mad1-bound C-Mad2.^84,108^ C-terminal to this region the coiled-coil of Mad1 is again interrupted by a flexible region (residues 585–596; termed the C-terminal hinge), which Alpha-Fold2 predicts to contain a novel short α-helix (Figures 5a-c and 8a).^93^ After this the coiled-coil resumes and ends in a globular head domain (Mad1^CTD^, residues 597–718) featuring a conserved RWD-fold (Ring, WD40, DEAD).^45,153^ This RWD domain consists of an antiparallel β-sheet of four β-strands, a short α-helix followed by two C-terminal α-helices (Figure 5c). The C-terminal hinge of Mad1 has recently been shown both in vitro and in vivo to facilitate a flexible fold-over of the Mad1^CTD^ back towards Mad1-bound Mad2.^49,109^ A combination of cryo-EM, cross-linking mass spectrometry and Alpha-Fold2 analyses provides a model of the catalytic scaffold where Mad1:C-Mad2 is in a folded state (Figure 8).^49^

### 5.2 The Bub1:Mad1 interaction

The crystal structure of Mad1^CTD^ bound to a phosphorylated Bub1^CD1^ peptide has recently been solved (Figure 8a).^45^ Bub1^CD1^ forms a single α-helix which lies diagonally across the largely hydrophobic interface of the coiled-coil stem of Mad1^CTD^. At the N-terminus of Bub1^CD1^ the pThr461 site at the start of the CD1 helix contacts the coiled-coil of one monomer and directly binds to Mad1 Arg617 of the conserved RLK motif (Figure 8a, panel 2). Specificity of this interaction is augmented by the ability of the pThr461 phosphate to stabilize the N-terminal α-helix dipole of Bub1^CD1^. The C-terminus of Bub1^CD1^ α-helix then contacts the opposite Mad1 monomer at the top of the coiled-coil and its adjoining head domain β-sheet and poses Bub1 C-terminal to the CD1 domain to extend across the top of the Mad1^CTD^ head domain.

### 5.3 The Mad1:Cdc20 interaction

The N-terminus of Cdc20 (Cdc20^N^) interacts with Mad1^CTD^ in a phosphorylation-dependent manner.^46,47,49^ Recently, it has been shown that phosphorylation of Mad1 Thr716 by Mps1, just two residues before the Mad1 C-terminus, directly modulates their interaction.^49^ Although no experimentally determined structure of Cdc20 bound to phosphorylated Mad1 exists, a combination of NMR, biophysics and AlphaFold2 analyses has generated a structural model of their interaction (Figure 8a).^49^ The N-terminal 35 residues of Cdc20 bind across Mad1^CTD^ using two regions with residual α-helicity (α1 and Box1). Cdc20 α1 lies diagonally across the coiled-coil, mimicking the Bub1^CD1^:Mad1^CTD^ interaction. With its N-terminus, α1 contacts one Mad1^CTD^ monomer at the conserved RLK site, and with its C-terminus, it contacts the top of the coiled-coil and adjoining head domain β-sheet of the adjacent monomer (Figure 8a, panel 3). The Box1 helix then binds to the β2 strand, β2/β3 loop adjacent to pThr716 (residues 652–661), and C-terminal α-helix, and positions its positively charged Arg/Lys residues to interact with the phosphothreonine site (Figure 8a, panel 2). The loop which connects Cdc20 α1 and Box1 α-helices also interacts with the conserved QYRL (residues 648–651) motif within the first β-strand of the head domain. This loop also contains four prolines which likely provide rigidity to orientate both helices.

### 5.4 Tripartite assembly of Bub1 and Cdc20 on Mad1^CTD^

Biophysical characterization has identified that only a single copy of Bub1^CD1^ and a single copy of Cdc20^N^ can bind to the Mad1^CTD^ homodimer.^45,49^ This unusual stoichiometry is most likely controlled by inherent asymmetry within Mad1^CTD^ in which the coiled-coil is bent with respect to the head domain and which makes only one side of the homodimer favorable for binding.^45,49^ Despite the binding sites of Bub1^CD1^ and Cdc20^N^ almost entirely overlapping, fluorescent anisotropy measurements have identified that Bub1^CD1^ and Cdc20^N^ bind concurrently to Mad1^CTD^.^49^ An experimentally validated AlphaFold2 model outlines how Mad1^CTD^ can accommodate both Bub1^CD1^ and Cdc20^N^ on opposite sides of the Mad1^CTD^ homodimer (Figure 8a).^49^ This study also reported pre-liminary evidence that Bub1 and Cdc20 binding to Mad1^CTD^ is not cooperative.^49^ This suggests that Cdc20^N^ and Bub1^CD1^ likely have preferential affinity for opposite sides of the asymmetric homodimer, a process which might be conferred by the additional interaction of the Cdc20 Box1 helix to the phosphorylated head domain of Mad1^CTD^. Further work is required to understand how the tripartite assembly of Cdc20 and Bub1 on Mad1^CTD^ is controlled.

### 5.5 The Bub1^ABBA^:Cdc20^WD40^ interaction

The ABBA motif of Bub1, binds to the C-terminal WD40 β-propeller of Cdc20, specifically between blades two and three.^197^ This interaction likely determines how Cdc20 is targeted and repositioned at kinetochores during MCC assembly (discussed in Section 4.5).^5^ The parallel orientation of Cdc20^N^ and Bub1^CD1^ on Mad1^CTD^ is thus optimally positioned to be compatible with the Bub1^ABBA^: Cdc20^WD40^ interaction (Figure 8a). Additionally, the dual anchorage of both Bub1 and Cdc20 to each other and to Mad1^CTD^ may be important for enhancing the stable assembly of the scaffold as the interaction of Bub1^CD1^ and Cdc20^N^ with Mad1^CTD^ is relatively weak (low μM range).^45,47,49,152^

## 6 Insights Into The Molecular Basis Of Catalytic C-Mad2:Cdc20 Formation

As discussed in Section 3, a wealth of biochemical and cellular data have carefully dissected out contributors to catalytic Cdc20:C-Mad2 association. Placing these experiments in the context of the structural model for the catalytic scaffold provides what seems to be a near-complete understanding of how unattached kinetochores overcome the kinetic barrier to the C-Mad2:Cdc20 association to rapidly generate the MCC.

### 6.1 Tripartite assembly together with Mad1 fold-over delivers the Cdc20 MIM to O-Mad2

The structural model of the catalytic scaffold outlines the molecular mechanisms for how Bub1 and Mad1 would spatially constrain and reposition the MIM of Cdc20 near its binding site on Mad2. Key to this seems to be the ability of Mad1^CTD^ to flexibly fold-over which in the context of tripartite Bub1:Mad1:Cdc20 assembly poises Cdc20 MIM to bind to the β6 strand of Mad2 with which it pairs as an antiparallel β-sheet in the C-Mad2:Cdc20 complex (Figures 4a and 8a).

This model is supported by several key experiments and observations. The Cdc20 MIM would be positioned too far away from O-Mad2 in the context of Cdc20 bipartite anchorage to Bub1 and Mad1.^46,49,109^ Decreasing the distance between Box1 and the MIM of Cdc20 impairs catalysis of C-Mad2:Cdc20 formation in vitro.^109^ Maintaining flexibility within the C-terminal hinge which enables fold-over is essential for catalytic MCC assembly in vitro and SAC signaling in vivo.^109^ The importance of coupling fold-over and the spatial constraints imposed by tripartite Bub1: Cdc20:Mad1 assembly is also highlighted in experiments that identify that catalysis induced by the C-terminal Mad1 hinge is dependent upon an intact Mad1:C-Mad2 interaction with Bub1 CD1 and Cdc20 Box1.^109^ Additionally, the CD1 and ABBA motifs of Bub1 individually contribute to catalyzing in vitro C-Mad2:Cdc20 formation.^46^

### 6.2 Bipartite anchorage of Cdc20 may also unveil the Cdc20 MIM

It has been reported that the N-terminus of Cdc20 interacts with its C-terminal WD40 domain,^22,48^ as well as self-interaction within the N-terminus of Cdc20.^46^ In vitro MCC assembly assays also show that truncating the N-terminus or C-terminus on either side of the Cdc20 MIM promotes the formation of C-Mad2:Cdc20.^46^ This has led to the suggestion that Cdc20 may exist in an autoinhibited state whereby self-interaction masks the MIM of Cdc20 and not only impairs the ability of the Cdc20 MIM from inducing Mad2 conversion but impedes the ability of the Mad2 safety-belt from being able to latch on to it.^5,22,48^ The structure of the catalytic scaffold suggests bipartite anchorage of Cdc20^N^ to pMad1^CTD^ and Cdc20^WD40^ to Bub1^ABBA^ as a plausible mechanism for relieving self-interaction of Cdc20 to unveil its central MIM (Figure 8). However, whether Cdc20 self-interaction impairs MIM accessibility, and whether bipartite anchorage of Cdc20 in conjunction with Mad1^CTD^ fold-over acts to directly relieve Cdc20 autoinhibition remains unclear and requires further investigation.

It should be noted that when a minimal kinetochore-targeting region of Bub1 (residues 1–280), which does not contain the ABBA motif, is fused to Mad1 in vivo, the checkpoint is still robust, although impaired.^152^ This may suggest that the interaction of Cdc20^N^ to Mad1^CTD^ is a critical contributor to promoting MIM accessibility. However, it is also possible that the KEN or ABBA motifs in BubR1 could bind to Cdc20 to relieve Cdc20 autoinhibition instead. Bub1 and BubR1 self-dimerize, which is likely how BubR1 is targeted to kinetochores and repositioned for MCC formation.^149^ It would therefore be interesting to test if Bub1^1–280^, where the BubR1 dimerization domain is removed, could also sustain a robust checkpoint in vivo and if this leads to a further decrease in catalytic MCC assembly in vitro.

## 7 Concluding Remarks

We now have a structural model for the catalytic Bub1: Mad1:C-Mad2 scaffold which provides key insights into how Bub1 and Mad1:C-Mad2 target and locally concentrate O-Mad2 and Cdc20 (Figure 8). This model also demonstrates how the tripartite complex of Bub1:Mad1: Cdc20 likely relieves Cdc20 autoinhibition and orchestrates the arrangement of the MIM of Cdc20 relative to its binding site on Mad2. The catalytic scaffold centered on the Mad1 platform likely functions through a “floating-and-catch” mechanism,^46,109^ where dual-anchorage of Cdc20 in conjunction with flexible Mad1^CTD^ fold-over “stretches” the N-terminus of Cdc20 to unveil and situate the Cdc20 MIM for rapid entrapment by the Mad2 safety-belt.

How much Mad1^CTD^ undergoes fold-over from a “fully extended” state to facilitate the C-Mad2:Cdc20 interaction as compared to Mad1 being constitutively folded which then has some mobility within the C-terminal hinge has yet to be addressed. Future studies should investigate whether there are any other factors, such as phosphorylation, that promote or regulate the Mad1^CTD^ fold-over. It is also an outstanding challenge to understand how the MIM of Cdc20 directly induces the O-Mad2 to C-Mad2 transition. Our current model suggests that the Mad1:C-Mad2 platform can perform only one catalytic cycle at a time because only one Bub1 and Cdc20 can bind at the same time (Figure 8a). This raises the question of whether both O-Mad2 reaction sites on Mad1:C-Mad2 are utilized. Because the fold-over of Mad1^CTD^ is highly flexible it is likely that either site could be used and the presence of two O-Mad2 molecules could facilitate Cdc20 MIM entrapment. Another possibility is that remodeling of the Mad1:C-Mad2 platform might occur which could then alter the favorability of either O-Mad2 reaction site. This could be related to how Mad2 conversion is accelerated by O-Mad2 dimerized to the Mad1:C-Mad2 platform, as it has been suggested that dimerized O-Mad2 presents an intermediate state which is more susceptible to spontaneous conversion.^196^

In summary, our new mechanistic knowledge of how cells overcome the kinetic barrier to the Mad2:Cdc20 association to rapidly assemble the MCC now sets the stage for understanding how similar cellular control mechanisms operate. This notably includes other HORMA-domain-containing molecular switches. Further insights can also be obtained by investigating how the catalytic scaffold functions in organisms where the molecular interactions of checkpoint proteins differ. This would especially be interesting in *C. elegans* where Mad1 and Bub1 do not interact through the CD1 of Bub1.^5,48,166^
